# Accounting for deep soil carbon in tropical forest conservation payments

**DOI:** 10.1038/s41598-024-65138-6

**Published:** 2024-07-22

**Authors:** Maja K. Sundqvist, Niles J. Hasselquist, Joel Jensen, Josefin Runesson, Rosa C. Goodman, E. Petter Axelsson, David Alloysius, Arvid Lindh, Ulrik Ilstedt, Francisco X. Aguilar

**Affiliations:** 1 Department of Forest Ecology and Management, Swedish University of Agricultural Sciences, 90183 Umeå, Sweden; 2 Department of Forest Biomaterials and Technology, Swedish University of Agricultural Sciences, 90183 Umeå, Sweden; 3 Department of Crop Production Ecology, Swedish University of Agricultural Sciences, 75007 Uppsala, Sweden; 4 Department of Wildlife, Fish and Environmental Studies, Swedish University of Agricultural Sciences, 90183 Umeå, Sweden; 5 Conservation and Environmental Management Division, Yayasan Sabah Group, P.O. Box 11623, 88817 Kota Kinabalu, Sabah Malaysia; 6 Department of Forest Economics, Swedish University of Agricultural Sciences, 90183 Umeå, Sweden

**Keywords:** Ecosystem services, Environmental economics, Environmental impact, Forest ecology, Ecosystem ecology

## Abstract

Secondary tropical forests are at the forefront of deforestation pressures. They store large amounts of carbon, which, if compensated for to avoid net emissions associated with conversion to non-forest uses, may help advance tropical forest conservation. We measured above- and below-ground carbon stocks down to 1 m soil depth across a secondary forest and in oil palm plantations in Malaysia. We calculated net carbon losses when converting secondary forests to oil palm plantations and estimated payments to avoid net emissions arising from land conversion to a 22-year oil palm rotation, based on land opportunity costs per hectare. We explored how estimates would vary between forests by also extracting carbon stock data for primary forest from the literature. When tree and soil carbon was accounted for, payments of US$18–51 tCO_2_^–1^ for secondary forests and US$14–40 tCO_2_^–1^ for primary forest would equal opportunity costs associated with oil palm plantations per hectare. If detailed assessments of soil carbon were not accounted for, payments to offset opportunity costs would need to be considerably higher for secondary forests (US$28–80 tCO_2_^–1^). These results show that assessment of carbon stocks down to 1 m soil depth in tropical forests can substantially influence the estimated value of avoided-emission payments.

## Introduction

The conservation of tropical forests is of utmost importance for reducing net greenhouse gas emissions in accordance with the Paris Agreement, the Glasgow Climate Pact^1^ and other global conservation initiatives. Deforestation and land degradation have been responsible for a large proportion of global greenhouse gas emissions^2,3^ and represent the second largest anthropogenic source of atmospheric carbon (C) after the combustion of fossil fuels^4^. In addition to reducing C stocks^5^ and increasing anthropogenic CO_2_ emissions^2,6^, tropical deforestation and land use change adversely impact biodiversity and a wide range of ecosystem services fundamental to human well-being^7,8^.

The expansion of oil palm (*Elaeis guineensis*) plantations has been one of the main drivers of deforestation in Southeast Asia^9^, largely occurring in tropical lowlands that are one of the world’s most biodiverse terrestrial ecosystems^10,11^. Oil palm plantations have been promoted as a pathway for rural economic development^12^ across Asia and in many developing economies due to high-yields, year-round income, and strong global markets^13^. Currently, oil palm is grown on *ca.* 20 million hectares of land globally^14^ with Indonesia and Malaysia accounting for 80% of the world’s palm oil production^10^. Oil palm agriculture is projected to continue on an expansionary trajectory in order to meet a growing demand for oil palm products^9,15^ with *ca.* 250 million hectares of land suitable for cultivation^16^. Balancing the trade-offs between forest conservation and financial benefits associated with land use change caused by oil palm agriculture remains a societal challenge.

As part of an attempt to reduce negative effects of the oil palm industry on environmental and ecosystem properties^17^, the Malaysian Sustainable Palm Oil certification was recently made mandatory by the Malaysian government. The certification is designed to promote sustainable palm oil production and practice, which enforces continued land use and hence avoids the use of land with high natural value. The majority of Malaysian oil palm cultivators are private estates but, smallholders also contribute to total oil palm yield^18^. The percentage of certified smallholders was initially low, but it has been recently shown that certification is associated with a higher profitability for smallholders, due to higher yields of fresh fruit bunches^18^. Further, the market imperfection caused by unpriced forest conservation and C emissions has contributed to creating a financial incentive to convert forested land to oil palm plantations^19^. A mechanism to correct this market imperfection is to provide conditional financial incentives for conservation^20^, with numerous national and international programs already offering payments to conserve high C stocks within tropical forests^21,22^. Assessing the additionality in conservation attributed to forest conservation payments has remained a challenge, in part due to the high opportunity costs of alternative land uses^23,24^, and the heterogeneous C stocks and sequestration rates across tropical landscapes^25–27^, including native unmanaged forests, native forests managed for timber, tree plantations, and agroforestry^28,29^. Nevertheless, research on this topic has contributed to assessing C gains during oil palm rotation periods, and thresholds for a C neutral conversion of different types of vegetation to oil palm^30,31^. However, data on deeper belowground C pools have been scarce in assessments of C dynamics associated with conversion from primary and secondary forest to oil palm plantations and generally only include estimates from the top 30 cm of soil^29,32,33^, which have been found to vary, but not show increasing or decreasing trends, over an oil palm rotation period^34^. Hence, important advances have been made to better account for uncertainty in C accounting, but little is still known about how accounting for C at deeper soil depths than 30 cm may influence estimates of C pricing.

**Figure 1:**
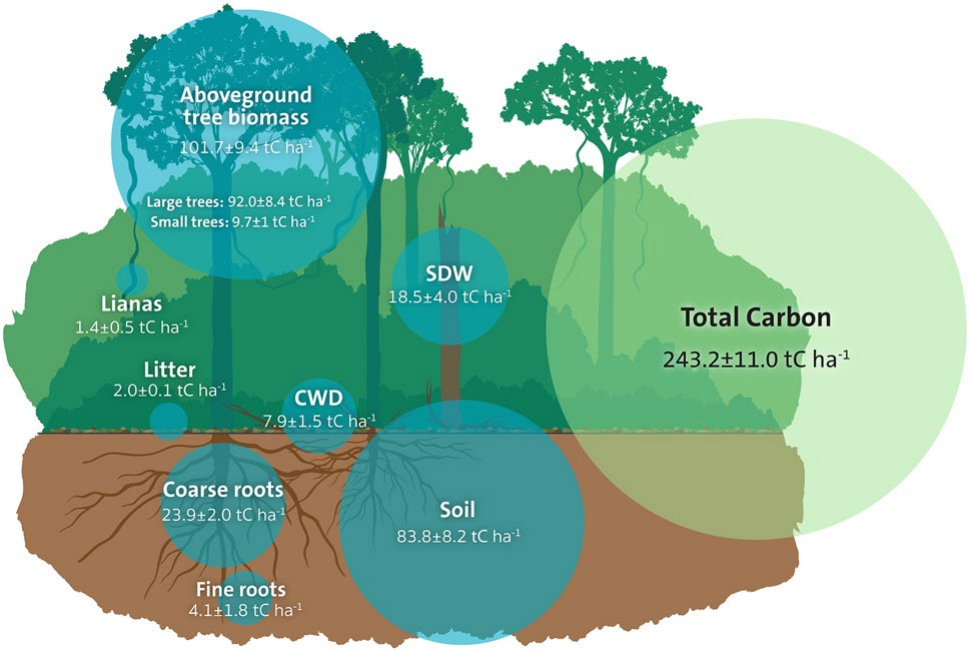
Carbon pools in a secondary forest. Total C stocks (tC ha^−1^), and the amount of C in above- and belowground C pools (tC ha^−1^; mean ± SE, N = 12), in a secondary dipterocarp forest ecosystem in Sabah, Borneo. The size of the circle for individual C pools corresponds to the amount of C in each pool. CWD = coarse woody debris, SDW = Standing dead wood. Illustration by Jerker Lokrantz, Azote.

**Figure 2:**
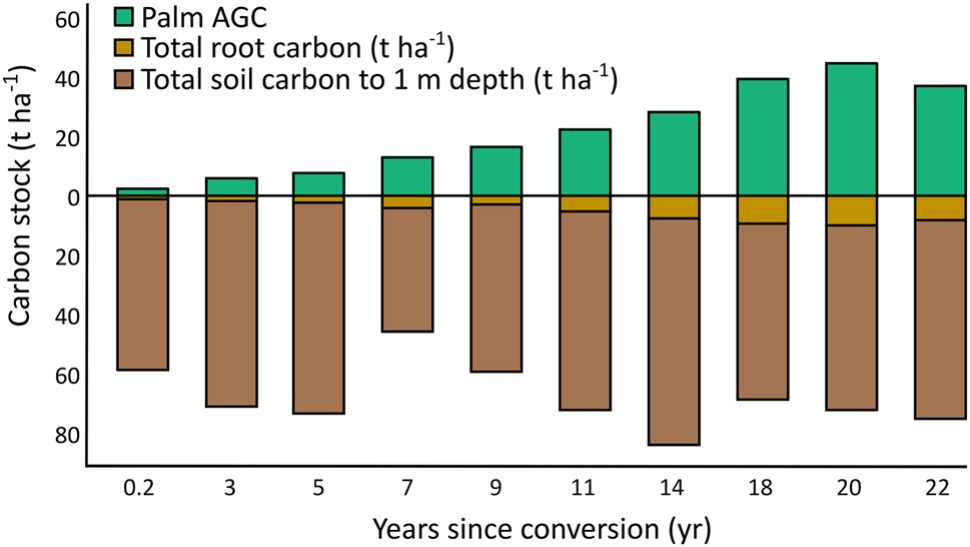
Above- and belowground carbon in oil palm plantations. Changes in aboveground oil palm C (Palm AGC) and soil organic C during a 22-yr rotation period of oil palm agriculture in Sabah Borneo. Each bar represents individual oil palm plantations that were converted at different times into oil palm production, except for the bar corresponding to 20 years since conversion which represents the avarage of two plantations.

This study quantifies soil C down to 1 m soil depth in estimated values of avoided-emission payments to promote tropical forest conservation, using study systems in Malaysia as a case study. To achieve this, we first conducted an assessment of total C lost per hectare during forest conversion to oil palm plantations in Sabah, Borneo by assessing C stocks above- and belowground, down to 1 m soil depth, per hectare in secondary forests^25^ and in oil palm plantations (Figs. 1, 2, Table S1, S2). We focused our field assessment on secondary forests because they represent *ca*. 70% of remaining tropical forest cover^14^, have a large potential for future C sequestration through restoration^35^, are representative of forests converted to oil palm plantations in Malaysia, and lie at the frontier of agricultural expansion facing the greatest risk of deforestation^36^. For comparisons between forests with different C stocks, we also extracted C stock data in primary forests from the available literature.

**Figure 3:**
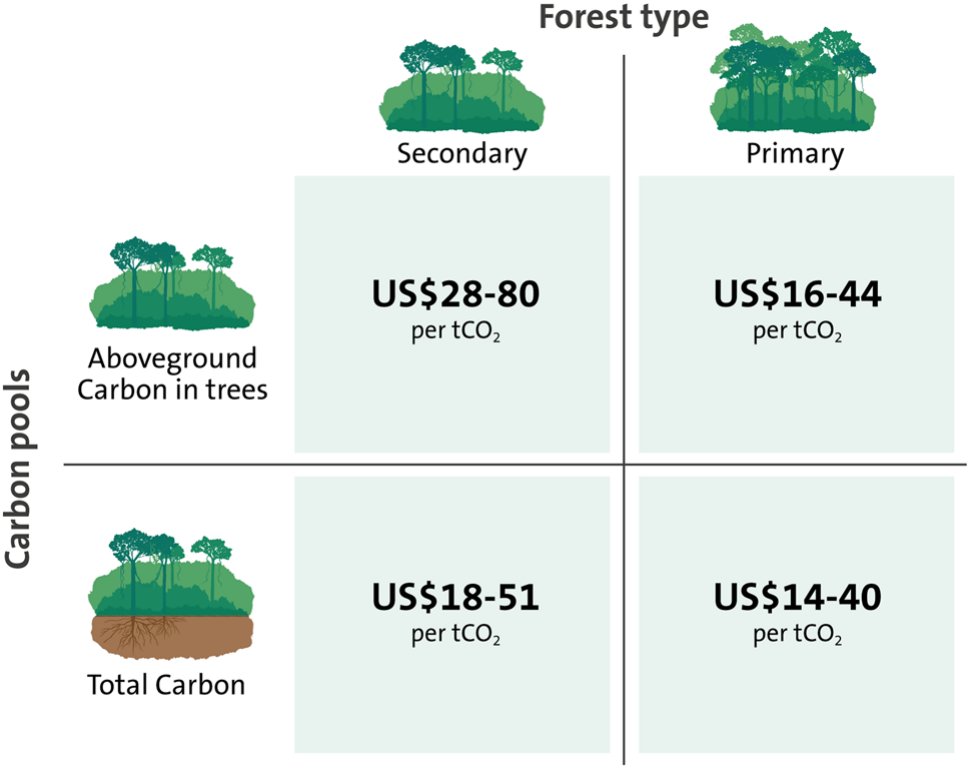
Carbon pricing estimates. Estimated C pricing for conservation of secondary and primary forests to offset opportunity costs of oil palm revenues (x-axis), including aboveground C stocks in tree biomass only, or total C corresponding to C in tree biomass and the soil (y-axis). Values represent the range of estimated C pricing needed to offset opportunity costs in a range of financial scenarios (see Methods and Table 1a) and using discount rates ranging between 2.5 and 10.0%. Illustration by Jerker Lokrantz, Azote.

We evaluated the financial value of oil palm agriculture (US$ ha^−1^) as a direct opportunity cost to forest conservation, by using recently published data on the net present value (NPV, 5% discount rate) of oil palm farming for uncertified independent smallholders in Malaysia^18^. We then inferred compensation payments for C that would offset forgone NPV of oil palm agriculture per hectare (Fig. 3, Table 1a–c). While our focus was to evaluate the role of soil C down to 1 m soil depth in C price estimates, we also explored how variability in NPV affected our estimates by calculating four additional financial scenarios. Within each scenario we included different discount rates (2.5–10%), to account for variation in risk preference levels, capital costs, and uncertainty for both smallholders and estates, and risk-adjusted revenues for fresh fruit bunches after stochastic simulations. Hence, our evaluation of C payments for avoided net C losses (US$ tCO_2_^–1^) included calculations that aimed to contextualize conditions for smallholders and larger estates. We assessed how estimates of C pricing per unit CO_2_ in a hectare vary by forest C stocks using data from primary and secondary forests; and whether soil C estimates down to 1m in-depth are included in payment calculations (Fig. 3). Hence, our estimates of C pricing per unit of carbon assume the same level of monetary compensation per hectare of forest regardless of type.

**Table 1:**
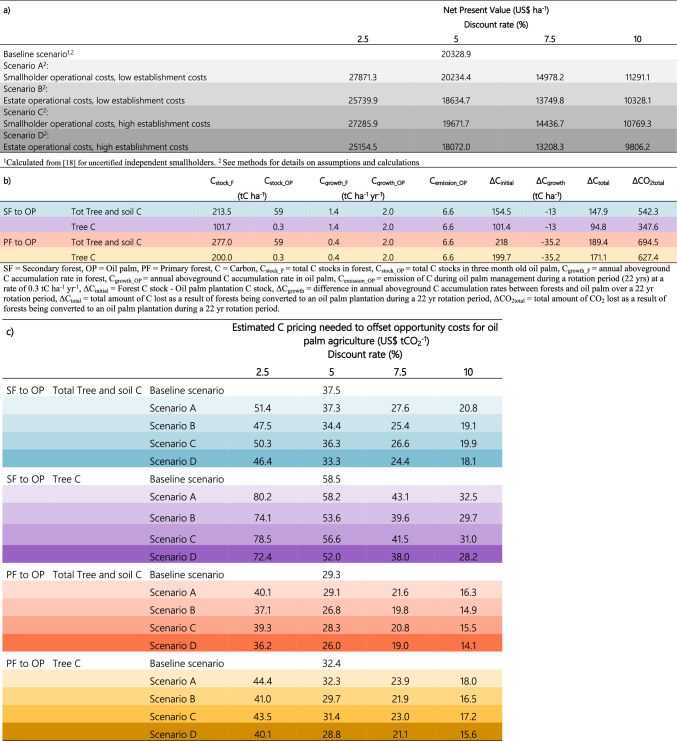
Calculating estimated carbon pricing. Net present values for different financial scenarios and discount rates **(a),** and C pools and amount of C lost during forest conversion to oil palm plantation over a 22 year rotation period **(b)**, used to calculate estimated C pricing needed to offset opportunity costs for oil palm agriculture in Sabah, Borneo, Malaysia **(c)**. See Methods for more details.

## Results and discussion

### Net C losses from secondary tropical forest conversion to oil palm plantation

Total C stock in the secondary forests was 243.2 ± 21.6 tC ha^−1^ (mean ± 95% confidence interval (CI), n = 12). Carbon in aboveground live tree biomass (101.7 ± 18.3 tC ha^−1^) and soil (83.8 ± 16.1 tC ha^−1^) were the two largest pools, representing 42 and 34% of forest C stock, respectively (Fig. 1). Our estimates of aboveground C fall within the range previously reported for secondary lowland dipterocarp forests in Southeast Asia (88.5–136 tC ha^−1^; Table S1), but are roughly 50% lower than what has previously been reported for primary dipterocarp forests in this region (e.g)^37^. Total belowground C (1 m depth) including root and soil C, corresponded to 46% of the forest C stock (111.8 ± 16.6 tC ha^−1^) reinforcing the importance of quantifying these pools when calculating total C stocks in these tropical forests.

Average total C stocks in oil palm plantations (92.3 ± 15.3 tC ha^−1^, n = 11) were *ca.* 40% of that observed in the secondary forest and ranged from 60.8 tC ha^−1^ in the youngest plantations to 116.7 tC ha^−1^ in older plantations (Table S2). Carbon in the aboveground tree biomass (21.9 ± 14.7 tC ha^−1^) was the C pool that differed the most between secondary forests and oil palm plantations, particularly in younger plantations (Fig. 2). In the oldest plantations (> 20 yrs) aboveground tree C was on average 66% lower compared to the secondary forest. We estimated an average annual aboveground oil palm C accumulation rate of 2.0 tC ha^−1^ yr^−1^, based on the regression model for oil palm plantation age (years) and oil palm biomass C in each plantation (R^2^ = 0.95; *p* < 0.01). This is within the range previously reported in the area^38^ and roughly 35% and 500% greater than reported by Suarez et al.^39^ for secondary and primary rainforests in Asia, respectively. The total belowground C stock in oil palm was on average 68.4 ± 7.4 tC ha^−1^ (Fig. 2) and soil C was on average 25% lower in oil palm plantations compared to secondary forests, consistent with previous studies in the region^32,33^. Further, there was variability in soil C stocks with time since conversion but we observed no increase or decrease in this stock across the different ages of oil palm plantations (R^2^ = 0.19; p = 0.20, n = 10), which is consistent with past reports down to 30 cm soil depth^34^.

Soil pH (1 m depth) was similar between the secondary forest (4.1 ± 0.2) and oil palm plantations (4.3 ± 0.2). Likewise, the average soil texture is similar in both systems (secondary forest: Clay: 30.8 ± 5.0%; Silt: 29.6 ± 4.8%; Sand: 39.6 ± 8.5% and oil palm: Clay: 27.8 ± 4.7%; Silt: 29.8 ± 4.0%; Sand: 42.5 ± 7.6%; Table S3). This equals to Clay loam when averaged down to 1 m depth.

### Payments to avoid net C emissions and promote secondary forest conservation

#### Carbon in secondary forest

Previous calculations of the potential revenues from oil palm plantations have ranged from US$4000 to US$29,000 ha^−1^^13,18,25^. In a recent study of independent smallholders in Malaysia, the NPV for uncertified smallholders was calculated as US$91,017.8 for 3.94 ha over a 25-year rotation, at a 5% discount rate with fertilization associated with the main costs^18^. We used this estimate to calculate a baseline NPV of US$20,329 ha^−1^ over a 22-year rotation period for our plots (Table 1a, baseline scenario). Based on C stocks in live tree biomass and soil per hectare, and assuming aboveground growth of 1.4 tC ha^−1^ yr^−1^ for secondary forests^39,40^, the conversion of secondary forest to oil palm plantation resulted in an average net loss of 147.9 tC ha^−1^ during a 22-year rotation period (Table 1b). We included C in live tree biomass in aboveground C stocks to be conservative in our estimates given how these stocks can vary greatly among secondary forests in this region, and to further allow comparability with previously published data from primary forest^37,41^. The conversion of secondary forest to oil palm plantation resulted in a net loss of 94.8 tC ha^−1^ from a reduction in aboveground tree biomass during the same rotation period (Table 1b). To financially support the conservation of secondary forests—by paying for avoided net C losses at a level that offsets the opportunity cost of forgone rents from oil palm agriculture—the estimated C pricing for our baseline scenario would have to be US$37.5 tCO_2_^–1^ when C stocks in tree biomass and soil are considered, and US$58.5 tCO_2_^–1^ when only aboveground C in tree biomass is considered (Fig. 3, Table 1c).

We also conducted estimates of the NPV for smallholders over a 22-year rotation period, using discount rates ranging from 2.5 to 10%^27^, to capture differences in landowner preferences and opportunity costs at levels comparable to recent analyses examining tropical forest conservation payments. The average estimates of NPV ranged between US$11,291–27,871 ha^−1^ in a scenario assuming a revenue from timber sales during conversion of US$1326 ha^−1^, establishment costs of total of US$2287 ha^−1^ for independent smallholders divided over the first 3 years, thereafter US$543 in annual operational costs following the production of the first fresh fruit bunches (FFB)^42–44^. (See Material and Methods, Table 1a, Scenario A). The estimated C pricing for this scenario would have to be US$21–51 tCO_2_^–1^ when C stocks in tree biomass and soil are considered, and US$33–80 tCO_2_^–1^ when only aboveground C in tree biomass is considered (Table 1c, Scenario A). When considering an additional three alternative scenarios, with increased establishment costs and/or differences in management costs between smallholders and estates (Table 1a, Scenario B-D), we found that alternative C pricing under such different revenue levels from oil palm agriculture would range between US$18–48 tCO_2_^–1^ if C stocks in tree biomass and soil were considered, and US$28–74 tCO_2_^–1^ when only aboveground tree biomass was considered (Table 1c). Further, a risk-assessment of our estimated NPV, with risk-adjusted estimated gross benefits from FFB by 0.38SD simulated 400 times (100 times for each selected outcome), found *ca.* 37–40% of our estimated NPV values at a 5% discount rate to be below those in the baseline scenario (Fig. S1). Overall, this assessment revealed a range in NPV of *ca.* US$6,000–35,000 ha^−1^. Hence, the estimated C pricing across the lowest and highest NPV accounting for this risk would need to be approximately US$11 tCO_2_^–1^ and US$65 tCO_2_^–1^, respectively, when C stocks in tree biomass and soil were considered, and US$17 tCO_2_^–1^ and US$101 tCO_2_^–1^, respectively, when only aboveground tree biomass was considered.

#### Carbon in primary forest

To consider a range in C stocks among tropical forests in our estimates, we also calculated payments that would offset opportunity costs of conserving C equivalent of what is found in primary lowland dipterocarp forests from conversion to oil palm agriculture. Here, we assumed average aboveground biomass in primary forest of 200 tC ha^−1^^37,41^ and an aboveground growth rate of 0.4 tC ha^−1^ yr^−1^^39,40^. When using the same approach as for secondary forest, we calculated that C pricing of US$29 tCO_2_^–1^ would offset opportunity costs when conserving C stocks in tree biomass and soil, and US$32 tCO_2_^–1^ would offset opportunity costs when conserving C stocks in tree biomass only, for the baseline scenario where the NPV for independent smallholders was US$20,328 ha^−1^ over a 22-year rotation period (Table 1a–c). Further, for the scenario assuming NPV ranging US$11,291–27,871 ha^−1^ for smallholders, we calculated that C pricing of US$16–40 tCO_2_^–1^ would offset opportunity costs when conserving C stocks in tree biomass and soil, and US$18–44 tCO_2_^–1^ would offset opportunity costs when conserving C in tree biomass (Table 1a–c, Scenario A). For scenarios assuming less revenue from oil palm plantations, this C pricing would range between US$14–39 tCO_2_^–1^ when C stocks in trees and soil was considered, and US$16–44 tCO_2_^–1^ when only C in tree biomass was included. Our estimated payment needed per unit mass of C is thus lower for primary than secondary forests since primary forests store considerably more aboveground tree biomass C per hectare than secondary forests (Table S1). Accounting for the risk-adjusted assessment in NPV values (Fig. S1), the estimated C pricing across the lowest and highest NPV would need to be approximately US$9 tCO_2_^–1^ and US$50 tCO_2_^–1^, respectively, when C stocks in tree biomass and soil are considered, and US$10 tCO_2_^–1^ and US$56 tCO_2_^–1^, respectively, when only C stocks in aboveground tree biomass was considered.

#### Soil carbon assessments, carbon pricing levels and conservation

Our findings show major differences in the estimates of financial compensation per avoided tCO_2_ when assessments of forest soil C stocks to 1 m depth are considered (Fig. 3, Table 1c). In several of our scenarios, the estimated C pricing needed to offset opportunity costs based on aboveground live tree C stocks in secondary forests are higher than C prices traded in the European Emission Allowances over 2018–2020 (US$7.5–29 tCO_2_^–1^)^45^, through California cap-and-trade auctions (US$15–17 tCO_2_^–1^) in 2019^46^, or projected global level C prices needed to protect 50% of investible tropical C sites globally (US$16 tCO_2_^–1^), and in the Asia–Pacific region (US$7.1 tCO_2_^–1^)^27^. However, some of our scenarios are more in line with what has previously been reported for pricing needed to overcome opportunity costs of conservation with respect to timber from primary forest and oil palm plantations in Southeast Asia (US$46–48 tCO_2_^–1^, at a discount rate of 10%)^25^ and the break-even US$35–51 tCO_2_^–1^ price to equal profits associated with rubber plantations in the region^26^. While many of the price estimates presented here and in other studies^25,26^ are higher than prevalent levels trading in C markets (about US$2.5–15 tCO_2_^–1^ for nature-based credits, nominal terms 2022–2023), they are more closely aligned with estimates derived from the social costs of C^47^ including a recent meta-analysis setting it at an average of US$55 per tCO_2_^48^.

When accounting for changes in belowground C pools after forest conversion, our study reinforces previous reports^32,33^ showing that soil C is on average 25% lower in oil palm plantations compared to secondary forests. Including soil C losses from conversion of secondary forest to oil palm down to 1 m soil depth in our estimates drastically reduced the estimated price per tCO_2_^–1^ needed to offset opportunity costs for secondary forests. Further, when such soil C losses were taken into account, the difference between C prices estimated to be needed to prevent conversion of primary and secondary forests was narrowed. While soil C estimates have been included in previous studies on C payments, e.g.^25,27^, these have often been based on coarser estimates and at a much shallower soil depth. Further, C pricing clearly varies depending on a number of factors such as the risks associated with oil palm agriculture (e.g. variation in production and price for fresh fruit bunches, costs of fertilizers and policy), and assumptions made about establishment, transaction and operational costs for oil palm plantations (e.g)^18,49^. Our study highlights that C prices per tCO_2_^–1^ needed to offset opportunity costs vary depending on the pools that are included in ecosystem estimates. We recognize that there are several reasons why the pool of C within deep soil layers have not been, or may not be, included in C pricing estimates, such as data scarcity, and difficulty of sampling. Nevertheless, our findings demonstrate that accounting for deeper soil C, as well as the overall variation in C stocks across forests, may strongly affect estimated C pricing needed to balance opportunity costs in avoided-emission payments poised to promote tropical forest conservation.

#### Implications for tropical forest conservation

Our estimates of C pricing needed to offset land opportunity costs are clearly different when comparing between C stocks found in secondary and primary forests, and when soil C stocks is or is not included in our estimates. However, we emphasize the constraints in our estimates, and also the context-specific nature of our results. The C stocks we measured in secondary forests are likely most representative of those in forests converted to oil palm plantations in Malaysia, and values including C stocks and land opportunity costs vary due to a number of site-specific factors such as accessibility, distance to markets, topography, soil fertility, climate, management practices, the risks associated with the market for oil palm and other relevant cash crops. For instance, current sustainability certification and policy in Malaysia enforces continued land use for oil palm and that the use of land with high natural value is avoided^18^. Hence, our comparison between primary and secondary forest was solely conducted based on the role of variation in C stocks. In addition, due to the scarcity of data on C accumulation rates including soil C down to 1 m depth in these systems, our estimated C prices accounts for soil C lost during conversion from forest to oil palm in half of our scenarios, but uses only aboveground annual C accumulation rates for primary and secondary forest and oil palm plantation in all scenarios. Further, we used nearby estimates of aboveground annual growth rates in secondary forests^39,40^. However, growth rates are highly variable in secondary forests^50,51^ and restoration or improved management could plausibly double annual aboveground growth^52^. Furthermore, the opportunity cost used in our calculations is based on the conversion of forests to oil palm plantations in Malaysia. Although our own calculations for smallholders (Table 1a; Scenario A, C) are comparable with recent calculations for Malaysia (Table 1a; baseline scenario calculated from^18^), these costs can vary greatly even within south-east Asia (e.g.^18,49^), with risks associated with oil palm farming such as the production and price of FFB (Fig. S1), and differ for other land use changes such as for rubber or eucalyptus plantations.

We assessed several scenarios for NPVs and our estimates assumed that all practices, including timber harvesting are legal. We do not consider potential costs associated with non-compliance such as any potential fines for engaging in illegal practices. Finding sustainable ways to deter tropical deforestation and land degradation is an urgent and challenging task. Hence, future work that assesses risks in greater detail (e.g.^53^) for oil palm farming, along with enhanced soil C stocks estimated at deeper depths, can improve the understanding and potential of C prices needed to offset opportunity costs under a much wider range of socio-economic and ecological scenarios than those presented in this study. It is important to mention that programs implemented to compensate landowners for avoided C emissions involve costs associated with the setting up and running of payment operations including monitoring and contractual compliance^54^. These transaction costs are very contextual with large variability often driven by administrative arrangements. Arguably, the biggest transaction costs have been found in environmental payment programs that require the creation of an entire new contractual system, as compared to one that is an add-on to existing commodities. Specific to payments for forest conservation, Wunder and Albán^55^ point to transaction costs ranging between 17 and 25% of total operational costs when accounting for program monitoring, promotion, certification, and administration—that did not include monitoring for soil C stocks. In sum, high transaction costs can pose a major challenge to the effective implementation of conservation payment programs. Finally, mechanisms focused on providing financial incentives to prevent forest losses on a commoditized C price have not been widely effective in promoting conservation to-date^27^. Nevertheless, our results show that estimates of C pricing needed to balance opportunity costs per unit area may vary less among forests when aboveground C stocks and belowground C stocks down to 1 m depth are considered.

The vast majority of tropical forests have experienced some type of anthropogenic disturbance and there is a growing awareness of the need to conserve and restore these forests^56^. During the past decade there has been a number of international declarations calling for forest conservation and restoration (*i.e.*, Bonn Challenge, New York Declaration on Forests, and the 2030 Agenda Sustainable Development Goals) and the United Nations has declared 2021–2030 as the decade for ecosystem restoration. Current agreements on provision of funding for vulnerable countries to cope with loss and damage caused by climate change stress the need to create mechanisms for a global transformation to a low-carbon economy, and to halt and reverse forest loss and land degradation by 2030 through voluntary conservation and compensation mechanisms^57–59^. Allocating limited financial resources needed to meet these ambitious goals will be challenging and needs to be efficient. The mechanisms focused on providing financial incentives to prevent forest losses on a commoditized C price have not been, and may not be, effective in promoting conservation. It has been argued that unless C prices increase other conservation interventions need to be implemented^27^, and they need to be accompanied by other efforts such as those enhancing human and social capital. Further, certification of sustainable oil palm agriculture in Malaysia associated with a higher profitability for smallholders^18^ may contribute to increased certification among independent smallholders.

Taken together with the recognized importance of secondary tropical forests for climate mitigation^60^ as well as biodiversity^61,62^, conserving remaining secondary forests may be an efficient use of limited financial funds to protect natural values^63^. Old-growth tropical forests often store more C than secondary and logged forest, yet old-growth forests represent only 30% of the entire forested area in the tropics and much of its acreage is already under a protected category^64^. Our estimates, based on net present value of oil palm agriculture per hectare and C stocks in forests per hectare, result in a higher price per ton of C for secondary forest (i.e. while the same amount of US$ would be paid to a land-owner per hectare of land regardless of forest type). Further, there may be a higher economic return on C credit payments to prevent deforestation of forests at the frontier of agricultural lands than, for instance, paying to engage in reforestation. Busch et al.^65^ estimate that compensation in the range of US$20–50 tCO_2_^–1^ could potentially avoid 55–108 GtCO_2_ emissions over 2020–2050 and, at US$20 tCO_2_^–1^, Malaysia is estimated as one of ten countries with the highest potential to reduce emissions from deforestation over this period. Hence, there are arguments in favor of protecting secondary forests and promoting their restoration in addition to protecting the remaining unprotected primary old-growth forests.

## Methods

### Study system

This study was conducted in the state of Sabah, in Malaysia’s northern Borneo. The secondary forest is situated ~ 8 km west of Luasong in the Sungai Tiagau Forest Reserve (4°28 N, 117°16 E). Oil palm plantations are located in the same general area but east and south of Luasong (Fig. S2). The forests within the Sungai Tiagau Forest Reserve were logged in the 1970s and a large part of the reserve burned during extensive wildfires in 1983/84^66^. Prior to logging activities and the wildfires in the 1980s, the area was characterized by *Dipterocarpaceae* dominated forests that are typical of lowland rainforests in this region^67^, whereas pioneer trees (notably *Macaranga* spp.) became the dominant tree species afterwards. Oil palm plantations are owned by Sabah Softwood Berhad that manages oil palm plantations under a rotation period of approximately 20 years. In this study, we focused on plantations that prior to conversion to oil palm agriculture were forested either as tree plantations or secondary forests except for the 3 year-old plantation which had replaced old oil palm plantations.

We installed and surveyed 12 study plots in the secondary forests on September–November 2017 and October 2018, and 11 plots in palm oil plantations during September–November 2018 (see Fig. S2 for information about the location of each study plot sampled within secondary forest and oil palm plantation). To account for the greater heterogeneity in the structure and biomass of secondary forests compared to oil palm plantations, we used study plots that were 60 × 60 m in secondary forest and 40 × 40 m in oil palm plantations. All plots were selected to represent similar soil properties (pH, clay, silt and sand content; Table S3), slope and aspect, and were located > 100 m from the nearest main road. Slope was estimated visually in oil palm, as well as aspect in both oil palm and secondary forest. Due to the greater heterogeneity in slope in the secondary forest, measurements were taken in the field to ensure plot placement on as comparable slopes as possible, with slope inclination ranging 10–36° across plots (Table S3). In secondary forests, the 12 plots were in an area where restoration through assisted natural regeneration and enrichment planting (with up to 300 native trees per ha) has occurred since 1998. In oil palm plantations, the 11 study plots were evenly placed along a chronosequence that represents roughly one rotation period, ranging in age from the youngest plantation (3 months since planting) to the oldest (22 yrs) plantation that was planted in 1996. These plots in secondary forest and oil palm plantation were used to collect data for above and belowground C pools^68,69^.

To determine total C losses as a result of conversion to oil palm plantations in this study system, we conducted detailed measurements of above- and belowground (1 m soil depth) C stocks in the secondary forest (Fig. 1) and across the chronosequence representing a rotation period of 22 years for first generation oil palm plantations (Fig. 2, Table S2). Most C stock measurements were conducted on subsamples within each plot, which were used to obtain an estimate of each C stock at the plot level, where the number of subsamples taken were assumed to be sufficient to account for spatial heterogeneity within each plot (see Data S1 and S2).

### Aboveground biomass and C measurements

Aboveground biomass was measured in oil palm plantations during September–November 2018 and used to calculate C in aboveground biomass. For plantations > 3 yrs old, we measured the height of all oil palm trees in each 40 × 40 m plot using a laser rangefinder (Nikon Forestry Pro Laser Rangefinder), and aboveground biomass for each oil palm plantation plot (kg) was calculated using the allometric Eq. 1, as per^70,71^ (Data S1):

1 $${\text{AGB}}_{{{\text{palm}}}} = { 71}.{797 } \times {\text{ H }}{-}{ 7}.0{872},$$

where AGB_palm_ is aboveground biomass of the oil palms (kg) and H is height of the oil palms (m). We multiplied aboveground biomass with the aboveground C content for oil palm plantations reported in^5^ (see Table S4) and scaled up these measurements to estimates of aboveground biomass in tC ha^−1^ per plot (Data S1).

In the recently established plantation, where oil palm trees do not have an obvious stem, we recorded the number and length of all individual fronds connected to each young oil palm in each 40 × 40 m plot. Then from a nearby oil palm nursery, we purchased five young oil palms from which we harvested 25 leaves and measured their individual length and dry weight and used that relationship to estimate the biomass of young oil palms, according to Eq. 2 (Data S1):

2 $${\text{Frond seedling dry weight }}\left( {\text{g}} \right) \, = { 32}0.{34 }*{\text{ frond seedling length }}\left( {\text{m}} \right) \, {-}{ 14}.{878},{\text{ R}}^{{2}} = \, 0.{9}0),$$

Given the strength of this relationship, we assume that the number of young oil palms and oil palm leaves used was sufficient for this estimate. Additionally, the 25 leaves from young oil palms were bulked into one composite sample and homogenized into smaller fragments. A subsample was taken from this composite sample to determine C content by dry combustion (Elementar Analysensysteme, Hanau, Germany, the Sepilok Forest Research Centre, Sandakan, Borneo, Malaysia). We multiplied aboveground biomass with the C content for young oil palms (Table S4) and scaled up these measurements to estimates of aboveground biomass in tC ha^−1^ per plot (Data S1).

To estimate aboveground biomass in the secondary forest, we measured the diameter at 130 cm from the ground (diameter-at-breast-height: dbh) of all large trees and lianas ≥ 10 cm within each 60 × 60 m plot. For trees with large buttresses, dbh was measured at 0.3 m above the highest buttress. The dbh for smaller trees and lianas (dbh: 10 cm >  ×  ≥ 5 cm) was measured in two randomly placed 10 × 10 m subplots within each larger 60 × 60 m plot in the secondary forest. Trees were identified to species or genus whenever possible. Liana biomass was estimated following Schnitzer et al.^72^, and tree aboveground biomass was estimated using allometric equations described in Basuki et al.^73^ (Table S5), calculated in R using the equations in Data S3–S4, and tree species data in Data S2, with wood density (g cm^−3^) derived from the Global Wood Density Database in 2019^74,75^. We used species-specific wood density values whenever available and genus-specific or a mean of family-specific values when this was not possible. Measurements of standing dead biomass were conducted using the same method as described above for large and smaller trees, with the exception that we also measured the height of standing dead trees to calculate volume and corrected for decay class as described in Chao et al.^76^. Missing height values for standing dead trees were estimated based on their diameter values using the linear relationship between measured standing dead tree diameter and height values.

Due to the often stochastic distribution of coarse woody debris, we measured the length and diameter of coarse woody debris (dead wood ≥ 2 cm in diameter) inside 1 m wide transects located along the outer edge of each 60 × 60 m plot in the secondary forest (2–4 transects per plot). Coarse woody debris that was possible to bring back to the laboratory was collected from within the transects and separated into decay class^76^, after which it was dried and weighed, and a subset was used for analyses of C content for each decay class. For large woody debris (*i.e.*, coarse woody debris that could not be brought to the laboratory), we collected a subsample for each decay class. The subsamples were brought back to the laboratory, dried and weighed for the determination of the respective dry mass fraction and analysis of C content. Biomass of large coarse woody debris was calculated by multiplying volume by the basal-area weighted mean wood density (*i.e.*, the mean wood density of all identified trees across plots weighted by their basal area) and corrected based on decay class^76^. The calculation of basal-area weighted mean wood density is represented in Eq. 3:

3 $$WD= \frac{{\sum }_{i}({\pi d}_{i}^{2}*{wd}_{i})}{{\sum }_{i}{\pi d}_{i}^{2}}$$

where *WD* is the basal-area weighted mean wood density across all plots, *d*_*i*_ is the diameter of the *i*^*th*^ tree and *wd*_*i*_ is the wood density of the *i*^*th*^ tree.

We calculated the amount of C (g) in large coarse woody debris by multiplying the volume (cm^3^), wood density (g cm^−3^) and C content (g g^−1^) for each sample. For each plot, we summed all coarse woody debris C (g) collected in the plot level transects, and used this to calculate total amount of C in coarse woody debris in tCha^−1^ per plot (Data S2). This was achieved by first dividing the total C (g) in course woody debris for each plot by the total area sampled (m^2^) which generated a value for course woody debris in gC/m^2^, and then multiplying this value with 0.01 (e.g. 10,000/1,000,000).

Fine litter (leaf litter and dead wood with diameter ≤ 2 cm) was collected in 0.5 × 0.5 m squares located in the center of 10 × 10 m subplots within each plot. Fine litter was collected in three subplots in each oil palm plantation plot, and in nine subplots in each secondary forest plot. Samples were dried to constant weight (85 °C; 3 days) for determination of total dry mass, and for calculation of total biomass of fine litter for each plot. A subsample of fine litter from each plot was analyzed for C content, and used to calculate average C content of litter from oil palm and secondary forest, respectively (Table S4). To calculate C in litter for each plot, we multiplied the biomass of litter in each subplot by the average C content of litter from oil palm and secondary forest, respectively, and calculated an estimated plot average in tC ha^−1^ (Table S4, Data S1–S2). In oil palm plantations only, we also determined the biomass of senesced palm fronds separately because of their large amount and heterogeneous distribution compared to fine litter. We measured the length of all senesced palm fronds in three to four randomly selected 10 × 10 m subplots within each plot to determine their biomass. The length and fresh weight was measured on 10 randomly selected senesced palm fronds within two of the plots before being dried to constant weight (85°C; 3 days). We then used this allometric equation to convert the length of senesced palm fronds measured in the field to dry biomass, based on the assumption that this relationship (R^2^ = 0.58, n = 10) is applicable across the study plots (Fig. S3). A subsample of the 10 senesced fronds collected in the field was taken for analyses of C content (Table S4). In each oil palm plantation plot, the average biomass of senesced fronds from the subplots, and C content in senesced fronds, was used to calculate the estimated C in senesced frond biomass in each plot in tC ha^−1^ (Data S1).

### Belowground measurements

In both the secondary forest and oil palm plantations, mineral soil samples were collected to a depth of 1 m. In each plot, nine soil samples were randomly collected at 0–10 cm and 10–20 cm depth using a metal cylinder (7.2 cm diameter, 10 cm long). Adjacent to each plot, a soil pit was dug to 1 m depth and deeper soil samples were collected with a metal cylinder (7.2 cm diameter, 5 cm long) horizontally into the soil at depths of 25, 35, 45, 65, 75 and 95 cm. These pits were dug immediately outside of the plots to minimize disturbance within the plots, as the plots were planned to be used for long-term measurements. An organic layer was only found in the secondary forests, which was sampled using a 7.2 cm diameter core and the depth recorded (cm).

In each plot, soil bulk density (g cm^-3^) was determined on three of the nine mineral soil cores collected at 0–10 cm and 10–20 cm depth and all soil cores > 20 cm depth (i.e. 25, 35, 45, 65, 75 and 95 cm), and soil dry mass was determined on the organic soil layer in the secondary forest (Data S1 and S2). All stones and roots were removed prior to drying each soil sample at 85°C until constant mass. For each soil sample, fine roots (< 2 mm) were dried and weighed, which allowed for the determination of fine root density (g cm^−3^). Biomass of coarse roots in the secondary forests was determined by assuming a root:shoot ratio of 0.235^77^, whereas a root:shoot ratio of 0.19 was used to determine the biomass of coarse roots in oil palm plantations^5^. For each plot, a subsample from all nine soil samples corresponding to organic, 0–10 cm and 10–20 cm depth were bulked to create one composite sample for further chemical analyses. Soil cores collected at depths ≥ 25 cm were bulked into two categories: one corresponding to 20–50 cm depth and consisting of samples collected at 25, 35 and 45 cm depth and the other corresponding to 50–100 cm and consisting of samples collected at 65, 75 and 95 cm depth. Soil texture was determined following the particle size distribution and soil pH was measured in a 1:2.5 ratio of soil to DI-water using a glass-calomel electrode. All soil analyses were conducted at the Forest Research Center Laboratory in Sepilok, Borneo, Malaysia.

For each plot, belowground biomass C pools were determined by multiplying the biomass for each C pool by its corresponding C content and extrapolating the data to tC ha^−1^ (Data S1–S2, Table S4). To determine mineral soil C, we multiplied the average bulk density from the different soil depths (*i.e.*, 0–10 cm, 10–20 cm, 20–50 cm, 50–100 cm) by the corresponding depth and C content for these layers (Table S4, Data S1–S2). Total C ( tC ha^−1^) was calculated by summarizing all biomass (live and dead) and soil C pools.

### Net present value of oil palm plantations

We used recently published data on net present value (NPV) from revenues and costs to independent smallholders at a discount rate of 5% as baseline against which we compared our own estimates (^18^; Table 1a, Baseline scenario). In Bok et al.^18^, NPV was based on oil palm plantations generating 17.88 ton fresh fruit bunches (FFB) ha^−1^ y^−1^ and a price of 187.79 US$ ton^−1^ FFB, where no harvests are conducted during the first 3 years of the oil palm cycle. Their NPV of 20,328 US$ ha^−1^ accounted for capital cost of acquiring land and operational costs (fertilizer, weedicide, seedlings, diesel gas and water), and assumed that smallholder farmers do not pay for any additional labor beyond that of the household’s^18^.

We estimated NPV based on revenues and costs generated over a 22-yr rotation period of oil palm using different annual discount rates, establishment costs and operational costs structured under four scenarios. This variation helped account for differences in landowner preferences and market conditions, and assessed the sensitivity of our estimates^25,27,78,79^. Our estimation of NPV (US$ ha^−1^) is presented in Eq. 4:

4 $${NPV}_{i,r}=\sum_{t=0}^{22} \frac{[{{Timber\, revenues}_{t} + (FFB\, production}_{t}\times Price\, of\, FFB)] -[{Establishment}_{t,i} + { ({Operation}_{t,i},)]}}{{(1+r)}^{t}},$$

where subscript *i* denotes the *i*th of our four scenarios; *r* denotes selected annualized discount rates; *t* captures cost in *t*h year of the 22-year rotation. Specific to our four scenarios, for the first two (A and B) we assumed establishment costs based on the lowest cost estimates for new plantations on normal soils in^44^, where costs correspond to 1355 US$ ha^−1^ in year 1474 US$ ha^−1^ year 2 and 457 US$ ha^−1^ year 3, adjusted for inflation using consumer price index 21% from 2007 to 2018 prices. The other two scenarios (C and D) assumed establishment costs based on the highest costs for new plantations on normal soils in^44^, corresponding to 1694 ha^−1^ in year 1610 ha^−1^ in year 2 and 593 ha^−1^ in year 3 (US$ ha^−1^). Scenarios A and C assumed operational costs of US$543 ha^−1^ yr^−1^ for smallholders, and B and D assumed operational costs of US$696 ha^−1^ yr^−1^ to represent higher costs likely to be incurred by estates as compared with smallholder farmers^43^ (Table S6). The NPV under the *i*th scenario was calculated with annual discount rates of 0.025, 0.05, 0.075, 0.10 for a total of 16 selected outcomes.

Revenue generated from oil palm was calculated by multiplying the weight of FFB (FFB production) by the average selling price (Price of FFB) established by the Malaysian oil palm board. Sabah Softwood Berhad provided data on the production of FFB over a wide range of different aged oil palm plantations, which allowed us to calculate annual FFB production for each year during the 22 yr rotation period (Fig. S4). We used the 2018 annual average FFB price of 107 US$ ton^−1^ FFB (Table S6)^80^. Annual oil palm operational costs encompassed values for upkeep (22%), fertilizer purchase and application (20%), FFB harvesting and collection (32%) and transportation (21%) based on Ismail et al.^43^ who used year 2000 prices, which we adjusted for inflation using a consumer price index of 45.89%^81^ to adjust prices to 2018. Hence, our evaluation of C payments for avoided net emissions included calculations for four scenarios that aimed to contextualize conditions for smallholders and estates (Table 1a). For all scenarios, we included an estimate of the benefits of sales of timber per ha^−1^ after land conversion (US$1326 ha^−1^; Data S2 in^42^ adjusted to 2018 US$ value).

We assume that the land is already owned, so there is no transaction cost in acquiring the land, and legal restrictions associated with land use changes are followed. In our estimates, we assume that unless land owners were compensated for conserving forests they would convert them to oil palm. Hence, the type of compensation payment we are investigating accounts for compensating landowners for not converting to oil palm agriculture based on avoided net emissions and the value that would need to be associated to them. Our calculations are intended to apply to first-generation oil palm plantations.

Further, and following the general analytical approach described in Tamba et al.^54^, we also introduced a measure of stochasticity when we risk-adjusted estimated gross benefits (production and price) from FFB. For this purpose, we introduced a random normally-distributed risk shifter ‘*k*’ with probability mean of ‘1’ and standard deviation of ‘0.38’ as per Eq. 5:

5 $${NPV}_{i,r}=\sum_{t=0}^{22} \frac{[{{Timber\, revenues}_{t} + (FFB\, production}_{t}\times Price\, of\, FFB \times k)] -[{Establishment}_{t,i} + { ({Operation}_{t,i},)]}}{{(1+r)}^{t}},$$

where subscript *i* denotes the *i*th of our four scenarios; *r* denotes selected annualized discount rates; *t* captures cost in *t*h year of the 22-year rotation; and* k* denotes a shifter to risk-adjust gross revenues from FFB . Risk-adjusted factors were run 100 for each selected outcome for a total of 1600 runs (Data S5). The standard deviation for risk-adjusting shifter ‘*k’* was derived from assessing annualized variability in estimates of FFB ha^−1^ yields and the annualized variance in price of FFB over the period 2015–2022.

### Carbon loss from conversion and a subsequent oil palm rotation period of 22 years

We applied linear regressions to assess how above- and belowground C stocks (Table S2) changed with time since conversion in oil palm plantation sites, using the statistical software Jamovi 2.3.28. The total amount of C lost as a result of forests being converted to an oil palm plantation during a 22 yr rotation period (ΔC_total;_ tC ha^−1^) was determined by [Eq. 6]:

6 $${\Delta C}_{total}={\Delta C}_{initial}+\sum_{t=1}^{22}{({\Delta C}_{growth})}_{t}+({{C}_{emission})}_{t}$$

where ΔC_initial_ denotes the initial loss of total C during the conversion of secondary forest to oil palm plantation (i.e., ΔC_initial_ = C_Stock_F_ − C_Stock_OP_); ΔC_growth_ (tC ha^−1^ yr^−1^) denotes the difference in annual aboveground C accumulation rates between forests and oil palm plantations; and C_emission_ represents annual C emissions associated with oil palm management during the entire rotation period (Data in Table 1b).

ΔC_initial_ for the conversion of a secondary forests to an oil palm plantation was calculated as the difference in C stocks (e.g. total soil C and/or aboveground tree biomass C stock) between the secondary forest (C_Stock_F_) and the 3-month old oil palm plantation measured in this study (C_Stock_OP_). Hence, ΔC_initial_ takes into account how much C that is lost during the conversion from forests to oil palm plantation, when accounting for either total soil C and aboveground tree biomass C stocks, or when only accounting for aboveground tree biomass C stock.

We calculated total C accumulation rate of 2.6 tC ha^−1^ yr^−1^ based on the regression analysis of plantation age (years) and total soil C to 1 m depth and oil palm biomass C (R^2^ = 0.79, *p* < 0.001, n = 11) in the oil palm chronosequence, but comparable data for primary and secondary forest are, to our knowledge, not available. Hence, in our estimate of ΔC_growth_ (tC ha^−1^ yr^−1^), we used 1.4 tC ha^−1^ yr^−1^ as the annual aboveground C accumulation rate in secondary forests based on a previous study in comparable secondary forest^40^ and values reported in older secondary forests in Asia^39^. Annual aboveground C accumulation rate in oil palm plantations was calculated as the annual aboveground oil palm C growth during the entire rotation period reported in this study (2.0 tC ha^−1^ yr^−1^), based on the regression analysis of oil palm plantation age (years) and oil palm biomass C (R^2^ = 0.95; *p* < 0.01).

For primary forest, we assumed an aboveground tree biomass C stock of 200 tC ha^−1^^37,45,82^, soil C stock of 77 tC ha^−1^^83^ and an annual aboveground C accumulation rate of 0.4 tC ha^−1^ yr^−1^^39^ for primary forests (Table 1b). We used 0.3t C ha^−1^ yr^−1^ as the emission of C during oil palm management^84^, which include diesel use for cultivation, transport and the application of fertilizer at a rate of 150–200 kg N ha^−1^ yr^−1^.

### Estimation of payments for avoided carbon emissions to prevent deforestation

We calculated the C pricing needed to conserve forest C by offsetting the foregone costs of not establishing an oil palm plantation by first converting ΔC_total_ to ΔCO_2,total_ by multiplying ΔC_total_ with the conversion constant 3.67 (e.g. mass ratio of CO_2_ and C; 44 g mol^−1^/12 g mol^−1^) (Table 1b). We then divided NPV (US$ ha^−1^) for oil palm plantation by ΔCO_2,total_ t ha^−1^ (e.g. the total amount of CO_2_ lost as a result of forests being converted to an oil palm plantation during a 22 yr rotation period; Table 1a,b), and expressed this as $US per tCO_2_ (Table 1c, Eq. 7). Hence, this estimated C pricing was calculated for both primary and secondary forests with only changes in aboveground tree C included and compared to C budgets including both tree and soil C, across our five scenarios and discount rates (Table 1). The goal was to assess how C pricing varies depending on the amount of C in forests being converted into oil palm agriculture, and whether soil C down to 1 m depth is included or excluded from the calculations.

7 $$Payment{\mkern 1mu} for{\mkern 1mu} avoided{\mkern 1mu} emissions\;({\text{US}}\$ /{\text{t}}) = \frac{{NPV\left( {\frac{{{\text{US}}\$ }}{{{\text{ha}}}}} \right)}}{{\Delta CO_{{2,total}} \left( {\frac{{\text{t}}}{{{\text{ha}}}}} \right)}}$$

### Supplementary Information

Supplementary Information 1.

Data S1.

Data S2.

Data S3.

Data S4.

Data S5.

## Data Availability

All data used in this study are included in the main figures and table, in the online supplementary material section.
