# Supplementary material for: Accounting for deep soil carbon in tropical forest conservation payments
Source: Sci Rep. 2024 Jul 22;14:16772. doi: 10.1038/s41598-024-65138-6 (PMC11263576; doi:10.1038/s41598-024-65138-6)
Supplement: Supplementary file 1 — Supplementary Information 1. [file 41598_2024_65138_MOESM1_ESM.pdf]

SUPPLEMENTARY MATERIAL

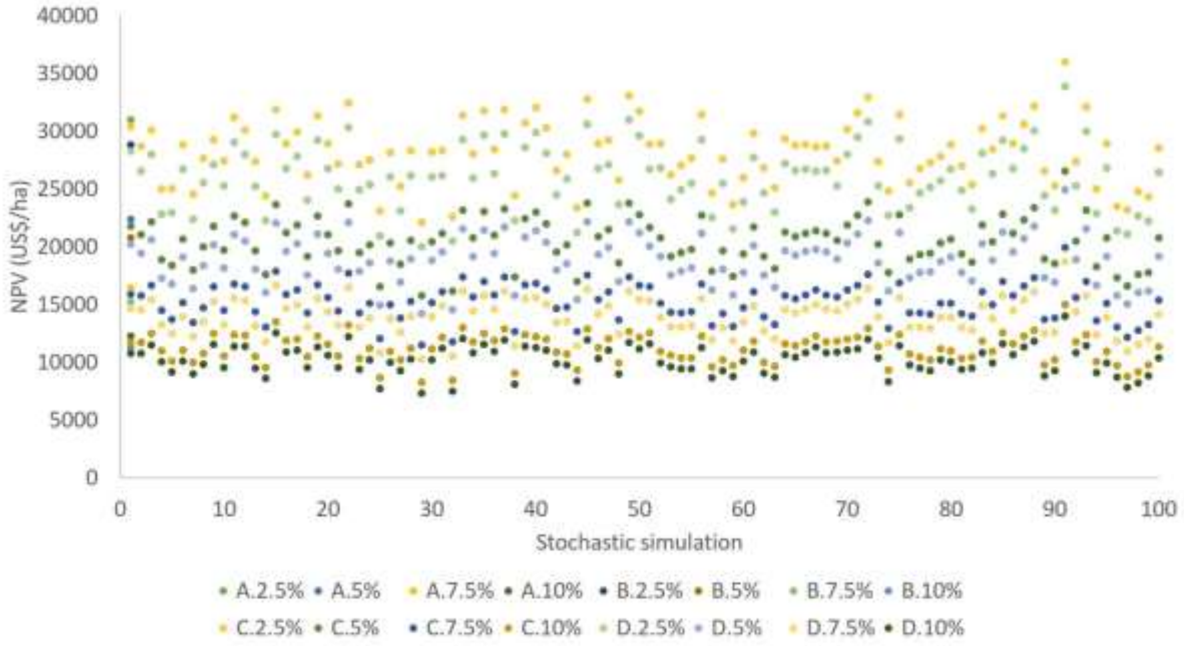

Figure S1. Net present value simulations. Scatter plot of simulated net present values of 22-year oil palm rotation under four selected establishment and operational cost scenarios (A, B, C, D) and annual discount rates (2.5%, 5.0%, 7.5% and 10%), with the inclusion of a random normally-distributed risk shifter in fresh fruit bunch revenues with average probability =1 and standard deviation = 0.38 (Equation 5).

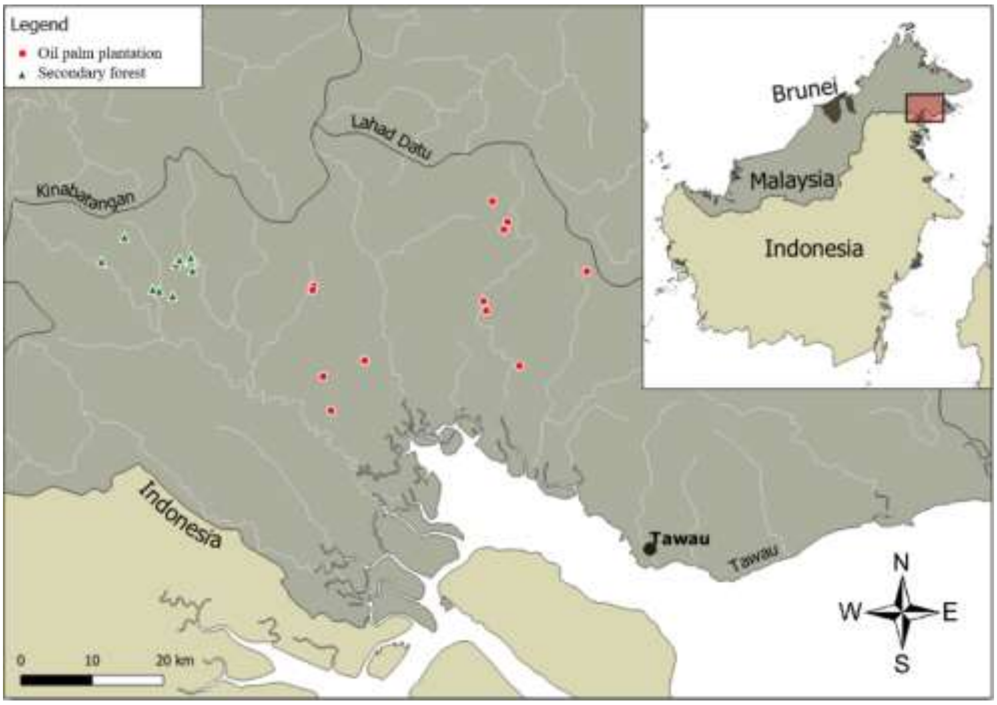

Figure S2. Study system. Location of study site within Sabah, Borneo as well as the location of the 12 secondary forest plots (green triangles) and 11 oil palm plantation plots (red circles) sampled in this study. The image is created in QGIS 3.10 A Coruña (https://gisenglish.geojamal.com/2019/11/download-qgis-310-coruna-nov-2019.html#more).

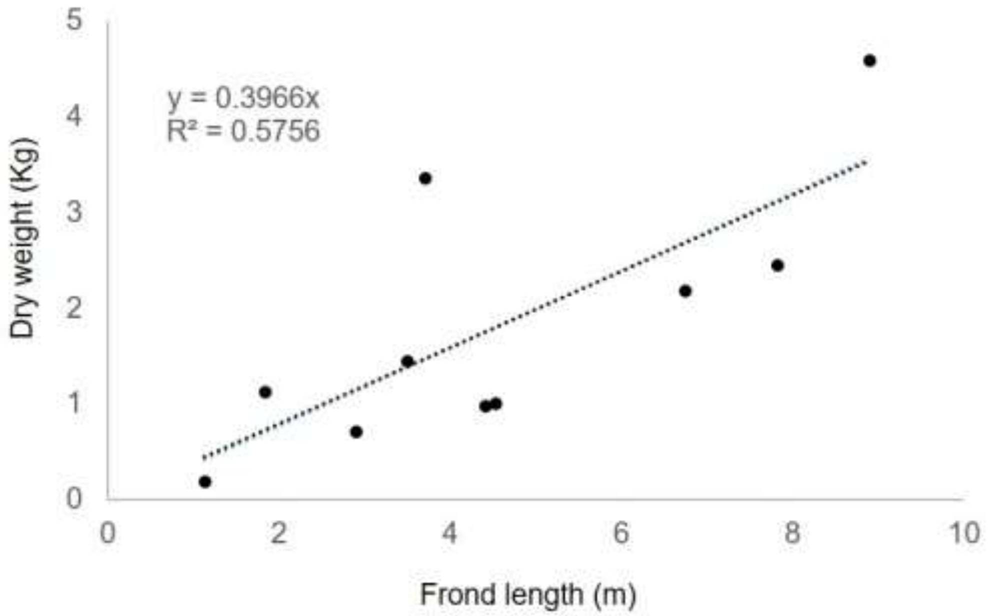

Figure S3. **Correlation between frond length and dry biomass of senesced palm leaves.** The slope of this relationship was used to convert measurements of senesced palm leaves in the field into dry biomass of senesced palm fronds. N = 10.

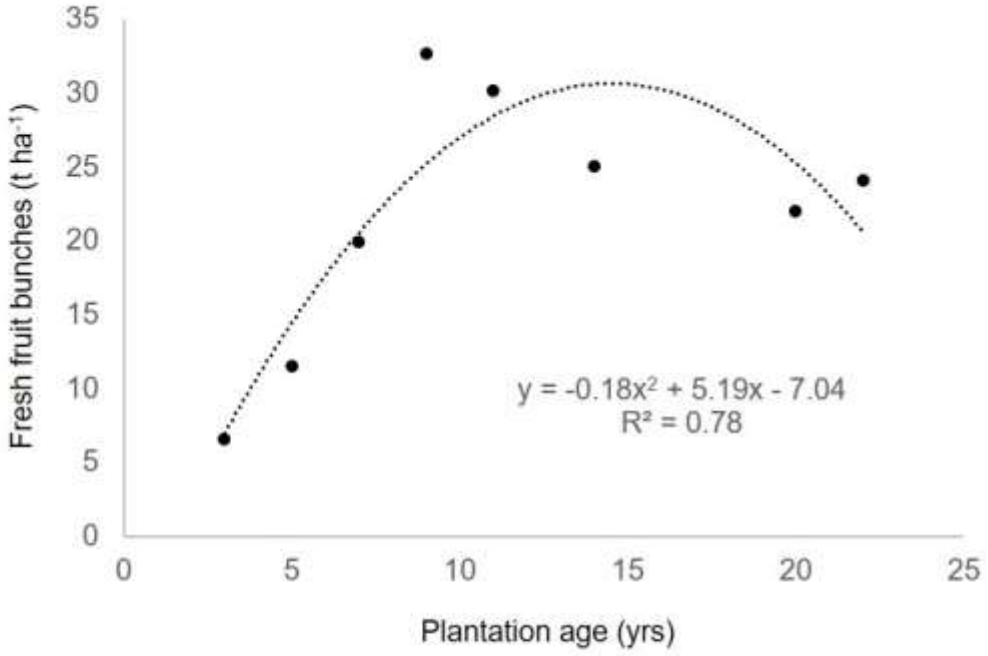

Figure S4. **Production of fresh fruit bunches (FFB) within different aged oil palm plantations**. This relationship was used to determine the production of FFB each year, which was used in calculating the net present value of oil palm production during a 22-year rotation period. N = 8.

Table S1. Carbon stocks (Mg C ha^{-1}) in old-growth primary forests and secondary humid tropical forests in Southeast Asia.

Forest Type	Study site	Mean SOC (Mg C ha^{-1})	Mean AGC (Mg C ha^{-1})	Diamater limit (cm dbh)	Reference
Primary Forest					
Sabah, Malayasia	Imbak Canyon FR		228.35 ± 77.25[f]	≥ 10 cm	Asner *et al.* 2018
Sabah, Malayasia	Maliau Basin FR		220.37 ± 69.32[f]	≥ 10 cm	Anser *et al.* 2018
Sabah, Malayasia	Danum Valley FR		207.36 ± 71.33[f]	≥10 cm	Anser *et al.* 2018
Peninsular, Malaysia	Pasoh Forest Reserve	10.8[a]	201.5		Adachi *et al.* 2011
Penisular, Malaysia	Pason Forest Reserve		155	≥5cm	Okuda *et al.* 2004
Kalimantan, Indonesia	Gunung Palung National Park		215 ± 12.5[g]	≥10 cm	Paoli *et al.* 2008
Kalimantan, Indonesia	Barito Ulu research area		179.2	≥10 cm	Brearly *et al.* 2004
Sumatra, Indonesia	Jambi province	77.0 ± 4.5[c,g]	159.7 ± 11.2[g]	≥2cm	Guillaume *et al.* 2018
Southeast Asia			197 ± 17.5[h]	≥10cm	Sullivan et al. 2017
Singapore	Bukit Timah Nature Reserve	77.5[d]	167.5	≥10cm	Ngo *et al.* 2013
Philippines	Surigao del Sur province	64.8[b]	193	≥ 19.5	Lasco *et al.* 2006
Secondary Forest					
Sabah, Malaysia	INIKEA	83.8 ± 18.1[d,h]	101.7 ± 18.7[h]	≥5cm	*This study*
Sabah, Malaysia	YSFMA[e]		89	≥5cm	Berry *et al.* 2010
Sabah, Malaysia	Malua Forest Reserve	39.6 ± 0.9[d,g]	91.9 ± 2.9[g]	≥10cm	Saner *et al.* 2012
Sabah, Malaysia	Sabah Biodiversity Experiment	58.2 ± 1.3[d,g]	136 ± 7.3[g]	≥10cm	Hector *et al.* 2011
Sabah, Malaysia	Class I and II forest reserve		100-110	≥10 cm	Asner *et al.* 2018
Peninsular, Malaysia	Pasoh Forest Reserve		137	≥5cm	Okuda *et al.* 2004
Kalimantan, Indonesia	Barito Ulu research area		132	≥10 cm	Brearley *et al.* 2004
Singapore	Bukit Timah Nature Reserve	103.9[d]	104.5	≥10cm	Ngo *et al.* 2013
Philippines	Surigao del Sur province	60.1[b]	139	≥19.5	Lasco *et al.* 2006

[a] SOC to a depth of 5cm
[b] SOC to a depth of 30 cm
[c] SOC to a depth of 50 cm
[d] SOC to a depth of 100 cm
[e] YSFMA = Yayasan Sabah Forest Management Area
[f] mean ± Standard deviation
[g] mean ± Standard error
[h] mean ± 95 % confidence interval

Table S2. Mean carbon stocks along a chronosequence of oil palm plantations that span an entire rotation period in Sabah, Malaysia.

	Oil Palm plantation									
Carbon pools (t C ha^{-1})	3 mo	3 yr	5 yr	7 yr	9 yr	11 yr	14 yr	18 yr	20 yr[a]	22 yr
Living Biomass										
Aboveground biomass[b]	0.3	4.9	5.8	9.9	13.1	19.6	26.8	37.4	43.5	35.8
Coarse root biomass[c]	0.06	0.89	1.07	1.81	2.39	3.57	4.88	6.83	7.93	6.53
Fine root biomass	0.8	0.9	1.5	2.2	0.8	1.7	2.6	2.6	2.1	1.9
Total living biomass	1.2	6.6	8.4	13.9	16.2	24.9	34.2	46.8	53.5	44.2
Dead Biomass										
Litter[d]	1.9	1.0	1.8	3.2	3.5	2.6	1.8	2.0	1.2	1.2
SOC										
0-10 cm	9.2	18.6	17.5	8.6	11.1	16.1	11.3	12.5	11.4	12.9
10-20 cm	10.1	14.0	11.3	6.2	8.1	10.8	6.3	7.8	6.9	10.6
20-50 cm	18.7	15.8	19.5	10.0	18.0	19.4	25.0	16.5	17.5	21.2
50-100 cm	19.7	21.1	22.6	16.9	19.2	20.5	33.7	22.6	26.4	22.1
0-100 cm	57.7	69.5	70.9	41.7	56.3	66.8	76.3	59.4	62.1	66.8
Aboveground pools[e]	2.2	5.9	7.6	13.1	16.6	22.2	28.5	39.5	44.7	37.0
Belowground pools[f]	58.6	71.3	73.5	45.7	59.5	72.1	83.8	68.8	72.1	75.2
Ratio AG/BG	0.04	0.08	0.10	0.29	0.28	0.31	0.34	0.57	0.62	0.49

[a]Average of two plots

[b]Aboveground biomass was determined using the allometric equation described in Asari et al. (2013) and a %C of 42.1% as reported in Kotowska et al. (2015). Aboveground biomass in the 3-month-old plantation was derived from an allometric equation converting leaf length to biomass (Fig. S3).

[c]Coarse root biomass was determined using a root:shoot ratio of 0.19 as found in Kotowska et al. (2015)

[d]includes both fine litter and fronds

[e]Aboveground pools include both aboveground biomass and litter

[f]Belowground pools include coarse and fine roots and SOC to 1 m depth

Table S3. Mean soil abiotic properties (a) along a chronosequence of oil palm plantations that span an entire rotation period (n = 11), and (b) in secondary forest plots (n= 12), in Sabah, Malaysia,

a)	Oil palm plantation									
	3 mo	3 yr	5 yr	7 yr	9 yr	11 yr	14 yr	18 yr	20 yr[a]	22 yr
pH										
0-10 cm	4.2	4.4	4.2	4.4	5.3	3.9	4.1	4.6	4.4	4.3
10-20 cm	4.2	4.5	4.2	4.3	5.1	3.6	3.9	4.2	4.0	4.2
20-50 cm	4.3	4.2	4.2	4.2	5.2	3.8	4.4	4.3	4.0	4.1
50-100 cm	4.4	4.2	4.1	4.2	5.8	4.2	4.4	4.3	4.3	4.5
0-100 cm	4.3	4.3	4.1	4.3	5.3	3.9	4.2	4.4	4.2	4.3
%Clay										
0-10 cm	25	19	26	15	25	37	19	34	32	17
10-20 cm	24	21	26	17	32	33	20	34	28	23
20-50 cm	13	19	23	17	46	27	26	41	29	23
50-100 cm	33	32	24	24	51	32	33	45	31	24
0-100 cm	24	23	25	18	39	32	25	39	30	22
%Silt										
0-10 cm	27	29	23	22	32	35	28	33	28	33
10-20 cm	28	26	24	22	33	38	24	33	36	34
20-50 cm	34	19	22	22	23	43	29	34	34	39
50-100 cm	31	22	20	17	29	40	25	32	35	37
0-100 cm	30	24	22	21	29	39	27	33	33	36
%Sand										
0-10 cm	47	52	51	63	43	28	53	33	39	50
10-20 cm	48	54	50	61	35	29	56	30	37	43
20-50 cm	53	62	54	62	32	30	45	25	37	37
50-100 cm	36	45	56	59	20	28	42	23	34	40
0-100 cm	46	53	53	61	32	29	49	28	37	42

[a]average of two plots

b)	Secondary forest											
	1	2	3	4	5	6	7	8	9	10	11	12
pH												
0-10 cm	3.94	4	4.1	4.18	3.67	3.66	3.63	4.07	3.77	3.66	3.66	4.23
10-20 cm	4.01	4.23	4.14	4.2	3.88	3.77	3.85	4.06	3.93	3.8	3.85	4.24
20-50 cm	4.07	4.32	4.39	4.37	3.67	3.82	3.81	4.25	4.58	4.06	3.92	3.94
50-100 cm	4.34	4.64	4.63	4.48	3.59	4.05	3.85	4.86	4.57	4.17	3.84	4.23
0-100 cm	4.09	4.30	4.32	4.31	3.70	3.82	3.78	4.31	4.21	3.92	3.82	4.16
%Clay												
0-10 cm	27.4	37.3	25.9	39.6	25.2	36.2	27.4	20.0	28.9	27.5	24.9	24.2
10-20 cm	30.7	27.6	27.2	43.7	27.5	37.1	30.4	25.6	23.7	27.4	27.3	26.3
20-50 cm	45.4	41.6	19.5	49,1	33.1	47.3	41.5	27.4	7.6	30.0	39.1	23.5
50-100 cm	43.3	37.2	13.3	53.5								
0-100 cm	36.70	35.9	21.5	46.5	28.6	40.2	33.1	24.4	20.1	28.3	30.4	24.7
%Silt												
0-10 cm	27.7	30.4	27.5	39.5	24.5	38.7	28.9	34.8	26.7	30.2	20.9	38.0
10-20 cm	26.0	28.2	28.0	38.2	25.6	31.2	39.1	42.5	22.2	27.5	19.8	39.4
20-50 cm	33.1	34.2	13.2	36.6	22.8	39.7	34.9	40.5	9.4	15.6	19.1	31.9
50-100 cm	36.4	38.1	11.2	32.4								
0-100 cm	30.8	32.7	19.9	36.7	24.3	36.5	34.3	39.3	19.5	24.5	20.0	36.4
%Sand												
0-10 cm	44.9	32.3	46.6	20.8	50.2	25.1	43.7	45.2	44.3	42.3	54.2	37.8
10-20 cm	43.4	44.2	44.9	18.0	47.0	31.7	30.5	31.8	54.0	45.0	52.9	34.3
20-50 cm	21.5	24.2	67.3	14.2	44.1	13.0	23.6	32.1	83.0	54.3	41.7	44.6
50-100 cm	20.3	24.7	75.5	14.1								
0-100 cm	32.5	31.4	58.6	16.8	47.1	23.3	32.6	36.4	60.4	47.2	49.6	38.9
Slope, °	10.4	10.0	25.1	24.7	20.5	18.7	33.7	24.8	36.1	21.1	19.2	27.8

Table S4. Carbon contents of biomass and soil in a secondary forest and oil palm plantation in northern Borneo.

Land use	Carbon pool	Decay class	Depth (cm)	Carbon content[a] (%)	Reference
Secondary Forest					
	Tree aboveground			47	Eggelston et al. 2006
	Litterfall			47.6	Both et al. (2017)
	Woody debris	1		49.8	*This study*
		2		47.4	*This study*
		3		34.7	*This study*
	Fine roots			35.8	*This study*
	Fine litter			46.0	*This study*
	Soil carbon		Organic layer	20.2	*This study*
			0-10	1.94 ± 0.53	*This study*
			10-20	0.94 ± 0.11	*This study*
			20-50	0.53 ± 0.13	*This study*
			50-100	0.42 ± 0.17	*This study*
Oil Palm plantation					
	Tree aboveground			42.1	Kotowska et al. (2015)
	Young fronds			47.8	*This study*[b]
	Coarse roots			40.4	Syahrinudin (2005)
	Fine roots			39.4	Kotowska et al. (2015)
	Fine litter			41.6	*This study*
	Frond litter			45.4	*This study*
	Soil carbon		0-10	0.95 ± 0.16	*This study*
			10-20	0.64 ± 0.12	*This study*
			20-50	0.44 ± 0.17	*This study*
			50-100	0.31 ± 0.08	*This study*

[a]Mean values, with mean ± 95% confidence intervals for soil carbon
[b]Data gathered from young oil palm individuals that we purchased from a nearby nursery.

Table S5. Allometric equations to determine biomass (kg dry weight) in a secondary forest in northern Borneo.

Use	Applied to	Equation	Source
Estimating tree BGB	Coarse roots (≥ 2 mm)	*Tree AGB * 0.235*	*Mokany (2006)*
Estimating dead biomass	Decay class 1	$v^a * (1.17 * \rho BA^b - 0.21)$	*Chao (2008)*
	Decay class 2	$v^a * (1.17 * \rho BA^b - 0.31)$	
	Decay class 3	$v^a * 0.29$	
Estimating tree AGB	*Dipterocarpus* spp.	$exp(-1.190 + 2.175 * log(diameter) + 0.082 * log(\rho^c)) * 1.023$	*Basuki (2009)*
	Hopea spp.	$exp(-1.708 + 2.335 * log(diameter) + 0.174 * log(\rho^c)) * 1.018$	
	Palaquium spp.	$exp(-0.723 + 2.145 * log(diameter) + 0.704 * log(\rho^c)) * 1.020$	
	Shorea spp.	$exp(-1.533 + 2.294 * log(diameter) + 0.560 * log(\rho^c)) * 1.030$	
	Other commercially valuable trees	$exp(-1.045 + 2.203 * log(diameter) + 0.639 * log(\rho^c)) * 1.057$	
	Fruit trees, pioneer trees, undefined	$exp(-0.744 + 2.188 * log(diameter) + 0.832 * log(\rho^c)) * 1.047$	
Esimating liana biomass	Lianas	$exp(-1.484+2.657*log(diameter))$	*Schnitzer (2014)*

[a] wood volume (cm^{-3})
[b] mean wood density of living trees weighted by basal area across all plots (g cm^{-3})
[c] species-, genus- or family-specific wood density (g cm^{-3})

Table S6. The predicted amount of fresh fruit bunch yield (FFB), revenues generated from FFB across 4-22 years since conversion to oil palm (a), and the price for FFB used to calculate revenues from FFB production across years, as well as operational cost and establishment costs across four scenarios (b).

a)

Year (22-year rotation)	Predicted ton FFB ha^{-1}	Revenues from FFB (USD ha^{-1})
4	10.845	1160.415
5	14.4226	1543.2182
6	17.643	1887.801
7	20.5062	2194.1634
8	23.0122	2462.3054
9	25.161	2692.227
10	26.9526	2883.9282
11	28.387	3037.409
12	29.4642	3152.6694
13	30.1842	3229.7094
14	30.547	3268.529
15	30.5526	3269.1282
16	30.201	3231.507
17	29.4922	3155.6654
18	28.4262	3041.6034
19	27.003	2889.321
20	25.2226	2698.8182
21	23.085	2470.095
22	20.5902	2203.1514

b)

	Benefits of timber sales (USD ha-1)[1]	Price of FFB (USD ton^{-1} FFB)[2]	Operational costs (USD ha^{-1} yr^{-1})[3]	Establishment costs (USD)[4]
Scenario A: Smallholder operational costs, low establishment costs	1326	107	543	2287
Scenario B: Estate operational costs, low establishment costs	1326	107	696	2287
Scenario C: Smallholder operational costs, high establishment costs	1326	107	543	2897
Scenario D: Estate operational costs, high establishment costs	1326	107	696	2897

[1]From Carrasco et al. 2017. [2]From Economics and industry development division, MPOB (2018). [3]From Ismael et al. (2009). [4]Total costs to maturity on normal soils in Wahit and Ismail (2009), adjusted to US$ in 2018. For Scenario A and B costs correspond to the lowest costs on normal soils and specifically to US$1355 in year 1, US$474 in year 2, and US$457 in year 3. For Scenario C and D, costs correspond to those estimated as the highest costs on normal soils, and specifically to US$1694 in year 1, US$610 in year 2 and US$592 in year 3.

References

Adachi, G., Ito, A., Ishida, A., Kadir, W.R., Ladpala, P. & Yamagata, Y. Carbon budget of tropical forests in Southeast Asia and the effects of deforestation: an approach using a process-based model and field measurements. *Biogeosciences* **8**, 2635-2647, doi:10.5194/bg-8-2635-2011 (2011).

Asner, G. P. *et al.* Mapped aboveground carbon stocks to advance forest conservation and recovery in Malaysian Borneo. *Biological Conservation* **217**, 289-310, doi:10.1016/j.biocon.2017.10.020 (2018).

Berry, N. J. *et al.* The high value of logged tropical forests: lessons from northern Borneo. *Biodiversity and Conservation* **19**, 985-997, doi:10.1007/s10531-010-9779-z (2010).

Both, S., Elias, D. M. O., Kritzler, U. H., Ostle, N. J. & Johnson, D. Land use not litter quality is a stronger driver of decomposition in hyperdiverse tropical forest. *Ecology and Evolution* **7**, 9307-9318, doi:10.1002/ece3.3460 (2017).

Brearley, F.Q., Prajadinata, S., Kidd, P.S, Proctor, J & Suriantata. Structure and floristics of an old secondary rain forest in Central Kalimantan, Indonesia, and a comparison with adjacent primary forest. *Forest Ecology and Management* **195**, 385-397, https://doi.org/10.1016/j.foreco.2004.02.048 (2004).

Carrasco, L.R., Webb, E.L., Symes, W.S., Koh, L.P. & Sodhi, N.S. Global economic trade-offs between wild nature and tropical agriculture. *PLoS Biology,* **15**(7), e2001657; https://doi.org/10.1371/journal.pbio.2001657 (2017)

Economics and Industry Development Division, MPOB. (2018) http://bepi.mpob.gov.my/index.php/en?option=com_content&view=article&id=906&Itemid=138 (2018).

Eggleston, H. S., Buendia, L., Miwa, K., Ngara, T., and Tanabe, K. IPCC guidelines for national greenhouse gas inventories. Volume 4: Agriculture, Forestry and Other Land Use, https://www.ipcc-nggip.iges.or.jp/public/2006gl/vol4.html (2006).

Guillaume, T. *et al.* Carbon costs and benefits of Indonesian rainforest conversion to plantations. *Nature Communications* **9**, 11, doi:10.1038/s41467-018-04755-y (2018).

Hector, A., et al. The Sabah Biodiversity Experiment: a long-term test of the role of tree diversity in restoring tropical forest structure and functioning. *Philosophical Transactions of the Royal Society B* **366**, 3303-3315, https://doi.org/10.1098/rstb.2011.0094 (2011)

Ismail, A., Simeh, M. A. & Noor, M. M. The production cost of oil palm fresh fruit bunches: The case of independent smallholders in Johor. *Oil Palm Industry Economic Journal* **3**, 1-7 (2003).

Kotowska, M. M., Leuschner, C., Triadiati, T., Meriem, S. & Hertel, D. Quantifying above- and belowground biomass carbon loss with forest conversion in tropical lowlands of Sumatra (Indonesia). *Global Change Biology* **21**, 3620-3634, doi:10.1111/gcb.12979 (2015).

Lasco, R.D., MacDicken, K.G., Pulhin, F. B., Guillermo, I. Q., Sales, R.F. & Cruz, R.V.O. Carbon stocks assessment of selectively logged Dipterocarp forest and wood processing mill in the Philippines. *Journal of Tropical Forest Science* **18**, 116-172 (2006).

Ngo, K.M. et al. Carbon stocks in primary and secondary tropical forests in Singapore. *Forest Ecology and Management* **296**, 81-89, https://doi.org/10.1016/j.foreco.2013.02.004 (2013).

Okuda, T. et al. Local variation of canopy structure in relation to soils and topography and the implications for species diversity in a rainforest of Peninsular Malaysia. Pp. 221-239 in Losos, E.C. & Leigh, E.G., Jr. (Eds) *Tropical Forest Diversity and Dynamism: Findings from a network of large-scale tropical forest plots*. University of Chicago Press, Chicago. (2004)

Paoli, G. D., Curran, L. M. & Slik, J. W. F. Soil nutrients affect spatial patterns of aboveground biomass and emergent tree density in southwestern Borneo. *Oecologia* **155**, 287-299, doi:10.1007/s00442-007-0906-9 (2008).

Saner, P., Loh, Y.Y., Ong, R.C. & Hector, A. Carbon stocks and fluxes in tropical lowland Dipterocarp rainforests in Sabah, Malaysian Borneo. *PLoS ONE* **7(1)**: e29642. doi:10.1371/journal.pone.0029642 (2012).

Sullivan, M. J. P. *et al.* Diversity and carbon storage across the tropical forest biome. *Scientific Reports* **7**, 12, doi:10.1038/srep39102 (2017).

Syahrinudin, S. The potential of oil palm and forest plantations for carbon sequestration on degraded land in Indonesia. *Ecology and Development Series* Vol. **28** (Cuvillier Verlag Göttingen, 2005)

Wahid, M. B. & Simeh, M. A. Issues related to production costs of palm oil in Malaysia. *Oil Palm Industry Economic Journal* **9**, 1-12 (2009).
