# Supplementary material for: Accounting for deep soil carbon in tropical forest conservation payments
Source: Sci Rep. 2024 Jul 22;14:16772. doi: 10.1038/s41598-024-65138-6 (PMC11263576; doi:10.1038/s41598-024-65138-6)
Supplement: Supplementary file 4 — Data S3. [file 41598_2024_65138_MOESM4_ESM.pdf]

Data S3 code - Small Trees

2023-10-27

```
#small trees data

smtrees <- read.csv("C:/Data S3.csv", header=TRUE, sep=";", colClasses=c("l
ifeform"="character"))


#where species-specific density exists, use that, otherwise genus-specific
density, otherwise family-specific density.

smtrees$wood_density <- ifelse(!is.na(smtrees$species_wood_density), smtree
s$species_wood_density,

                     ifelse(!is.na(smtrees$genus_wood_density), smtrees$gen
us_wood_density,

                            ifelse(!is.na(smtrees$family_wood_density), smt
rees$family_wood_density, NA)))


#replacing NAs with zeros

smtrees$species_wood_density[is.na(smtrees$species_wood_density)] <- 0

smtrees$genus_wood_density[is.na(smtrees$genus_wood_density)] <- 0

smtrees$family_wood_density[is.na(smtrees$family_wood_density)] <- 0


#convert diameter to basal area

smtrees$basal_area = pi*(smtrees$diameter)^2


#sum of basal area

sumba_smtree=sum(smtrees$basal_area, na.rm = TRUE)

sumba_smtree
```

```
## [1] 39361.23
```

```
getwd()
```

```
## [1] "C:/Users/jlen0002/OneDrive - Sveriges lantbruksuniversitet/lokalt/r
script/INIKEA"
```

```
#basal area weighted sum of basal area and wood density

sumwd_ba_smtree=sum(smtrees$basal_area*smtrees$wood_density, na.rm = TRUE)

sumwd_ba_smtree
```

```
## [1] 21376.36
```

```
#basal area weighted mean wood density

meanwd_bawt_smtree = sumwd_ba_smtree / sumba_smtree

meanwd_bawt_smtree
```

```
## [1] 0.5430815
```

```r
#mean wood density

meanwd_smtree <- mean(smtrees$wood_density, na.rm=TRUE)

meanwd_smtree
```

```
## [1] 0.5711256
```

```r
#for dead wood, calculate wood density using basal area weighted mean wood density assuming decay class 1

smtrees$wood_density <- ifelse(smtrees$lifeform=="dead", (1.17 * meanwd_bawt_smtree) - 0.21, smtrees$wood_density)


#allometric models for each genus described in Basuki 2009, otherwise "other commercial" or "mixed"

dipt_bas_model_3 <- exp(-1.190 + 2.175 * log(smtrees$diameter) + 0.082 * log(smtrees$wood_density)) * 1.023#dipterocarp

hope_bas_model_3 <- exp(-1.708 + 2.335 * log(smtrees$diameter) + 0.174 * log(smtrees$wood_density)) * 1.018#hopea

pala_bas_model_3 <- exp(-0.723 + 2.145 * log(smtrees$diameter) + 0.704 * log(smtrees$wood_density)) * 1.020#palaquium

shor_bas_model_3 <- exp(-1.533 + 2.294 * log(smtrees$diameter) + 0.560 * log(smtrees$wood_density)) * 1.030#shorea

comm_bas_model_3 <- exp(-1.045 + 2.203 * log(smtrees$diameter) + 0.639 * log(smtrees$wood_density)) * 1.057#other commercial

mixe_bas_model_3 <- exp(-0.744 + 2.188 * log(smtrees$diameter) + 0.832 * log(smtrees$wood_density)) * 1.047#mixed


#model for estimating liana biomass

liana_model <- exp(-1.484+2.657*log(smtrees$diameter))


#applying models for estimating living tree biomass

smtrees$tree_agb <- ifelse(smtrees$lifeform == "tree" & smtrees$genus == "Dipterocarpus", dipt_bas_model_3,

                        ifelse(smtrees$lifeform == "tree" & smtrees$genus == "Hopea", hope_bas_model_3,

                        ifelse(smtrees$lifeform == "tree" & smtrees$genus == "Shorea", shor_bas_model_3,

                        ifelse(smtrees$lifeform == "tree" & smtrees$genus == "Palaquium", pala_bas_model_3,

                        ifelse(smtrees$lifeform == "tree" & smtrees$species_group == "Dipterocarp", comm_bas_model_3,

                        ifelse(smtrees$lifeform == "tree" & smtrees$species_group == "Other commercial", comm_bas_model_3,

                        ifelse(smtrees$lifeform == "tree" & smtrees$species_group == "Fruit tree", mixe_bas_model_3,
```

```
                    ifelse(smtrees$lifeform == "tree" & smtrees$species_gro
up == "Pioneer", mixe_bas_model_3,
                    ifelse(smtrees$lifeform == "tree" & smtrees$species_gro
up == "Other", mixe_bas_model_3, NA)))))))))


#applying models for estimating liana biomass
smtrees$liana_agb <- ifelse(smtrees$lifeform=="liana", liana_model, NA)


#applying mixed model for estimating dead wood biomass
smtrees$dead_agb <- ifelse(smtrees$lifeform=="dead", mixe_bas_model_3, NA)


#calculating total AGB
smtrees$tot_small_biomass <- rowSums(smtrees[, c("liana_agb", "tree_agb", "
dead_agb")], na.rm = TRUE)


#carbon content
CC=0.47


#constant for carbon per hectare
constant_CC = (10000 / (20 * 10)) * CC


#aggregate plot data
plot_agb = aggregate(list(smtrees[c(17:20)]), by=list(smtrees$plot), sum, n
a.rm=TRUE)
colnames(plot_agb)[1] <- "Plot"


#calculate plot C
plot_C = plot_agb[, c(2:5)] / 1000 * constant_CC
plotsum_small = cbind(plot_agb[1], plot_C)


#write CSV
write.csv(plotsum_small,"C:/plotsum_small.csv")
```
