# Supplementary material for: Accounting for deep soil carbon in tropical forest conservation payments
Source: Sci Rep. 2024 Jul 22;14:16772. doi: 10.1038/s41598-024-65138-6 (PMC11263576; doi:10.1038/s41598-024-65138-6)
Supplement: Supplementary file 5 — Data S4. [file 41598_2024_65138_MOESM5_ESM.pdf]

Data S4 code - Large Trees

2023-10-27

```r
#large trees data
lgtrees<-read.csv("C:/Data S4.csv", header=TRUE, sep=";")


#where species-specific density exists, use that, otherwise genus-specific density, otherwise family-specific density
lgtrees$wood_density <- ifelse(!is.na(lgtrees$species_wood_density), lgtrees$species_wood_density,
                     ifelse(!is.na(lgtrees$genus_wood_density), lgtrees$genus_wood_density,
                            ifelse(!is.na(lgtrees$family_wood_density), lgtrees$family_wood_density, NA)))


#replacing NAs with zeros
lgtrees$species_wood_density[is.na(lgtrees$species_wood_density)] <- 0
lgtrees$genus_wood_density[is.na(lgtrees$genus_wood_density)] <- 0
lgtrees$family_wood_density[is.na(lgtrees$family_wood_density)] <- 0


#convert diameter to basal area
lgtrees$basal_area = pi*(lgtrees$diameter)^2


#sum of basal area
sumba=sum(lgtrees$basal_area, na.rm = TRUE)
sumba
```

```
## [1] 5333352
```

```r
#sum of basal area weighted wood density
sumwd_ba=sum(lgtrees$basal_area*lgtrees$wood_density, na.rm = TRUE)
sumwd_ba
```

```
## [1] 2713995
```

```r
#basal area weighted mean wood density
meanwd_bawt = sumwd_ba / sumba
meanwd_bawt
```

```
## [1] 0.5088723
```

```r
#mean wood density
meanwd <- mean(lgtrees$wood_density, na.rm=TRUE)
```

```
meanwd
```

```
## [1] 0.5694928
```

```
#allometric models for each genus described in Basuki 2009, otherwise "othe
r commercial" or "mixed"

dipt_bas_model_3 <- exp(-1.190 + 2.175 * log(lgtrees$diameter) + 0.082 * lo
g(lgtrees$wood_density)) * 1.023#dipterocarp

hope_bas_model_3 <- exp(-1.708 + 2.335 * log(lgtrees$diameter) + 0.174 * lo
g(lgtrees$wood_density)) * 1.018#hopea

pala_bas_model_3 <- exp(-0.723 + 2.145 * log(lgtrees$diameter) + 0.704 * lo
g(lgtrees$wood_density)) * 1.020#palaquium

shor_bas_model_3 <- exp(-1.533 + 2.294 * log(lgtrees$diameter) + 0.560 * lo
g(lgtrees$wood_density)) * 1.030#shorea

comm_bas_model_3 <- exp(-1.045 + 2.203 * log(lgtrees$diameter) + 0.639 * lo
g(lgtrees$wood_density)) * 1.057#other commercial

mixe_bas_model_3 <- exp(-0.744 + 2.188 * log(lgtrees$diameter) + 0.832 * lo
g(lgtrees$wood_density)) * 1.047#mixed


#snag height

estH <- lm(lgtrees$snag_height~lgtrees$diameter)#estimated dead wood height

lgtrees$snagH <- ifelse(lgtrees$lifeform=="dead" & lgtrees$snag_height > 0,
lgtrees$snag_height, NA)#measured dead wood height

lgtrees$snagHs <- ifelse(lgtrees$lifeform=="dead" & lgtrees$tree_ID == 213|
lgtrees$tree_ID==220|lgtrees$tree_ID==1188|lgtrees$tree_ID==321|lgtrees$tre
e_ID==2599|lgtrees$tree_ID==2708|lgtrees$tree_ID==2731|lgtrees$tree_ID==413
|lgtrees$tree_ID==484|lgtrees$tree_ID==487, estH$fitted.values, NA)#applyin
g estimated height to dead wood missing height

lgtrees$snagA <- ifelse(lgtrees$lifeform == "dead", (((lgtrees$diameter) ^
2) * pi), NA)#dead wood basal area (cm^2)


#dead wood volume

lgtrees$dead_wood_volumeH <- ifelse(lgtrees$snagH > 0, ((lgtrees$snagH * 10
0 * lgtrees$snagA)), NA) #dead wood volume (*100 to convert meters to cm to
get volume in cm3)

lgtrees$dead_wood_volumeHs <- ifelse(lgtrees$snagHs > 0, ((lgtrees$snagHs *
100 * lgtrees$snagA)), NA) #dead wood volume for dead wood with estimated h
eights

lgtrees$dead_wood_volume <- ifelse(!is.na(lgtrees$dead_wood_volumeH) & lgtr
ees$dead_wood_volumeH > 0, lgtrees$dead_wood_volumeH,

                                   ifelse(!is.na(lgtrees$dead_wood_volumeHs
) & lgtrees$dead_wood_volumeHs > 0, lgtrees$dead_wood_volumeHs, NA))


#applying model for estimating dead wood biomass with measured heights

lgtrees$dead_wood_mass <- ifelse(lgtrees$decay_class== "1", (((1.17 * meanw
d_bawt) - 0.21) * lgtrees$dead_wood_volumeH),
```

```
                              ifelse(lgtrees$decay_class== "2", (((1.17 * meanwd_bawt) - 0.31) * lgtrees$dead_wood_volumeH),

                                     ifelse(lgtrees$decay_class== "3", (0.29 * lgtrees$dead_wood_volumeH), NA)))


#applying model for estimating dead wood biomass with estimated heights

lgtrees$dead_wood_mass_est <- ifelse(lgtrees$decay_class== "1", (((1.17 * meanwd_bawt) - 0.21) * lgtrees$dead_wood_volumeHs),

                                 ifelse(lgtrees$decay_class== "2", (((1.17 * meanwd_bawt) - 0.31) * lgtrees$dead_wood_volumeHs),

                                        ifelse(lgtrees$decay_class== "3", (0.29 * lgtrees$dead_wood_volumeHs), NA)))#for dead wood with estimated heights


#model for estimating liana biomass

liana_model <- exp(-1.484+2.657*log(lgtrees$diameter))


#applying models for estimating living tree biomass

lgtrees$tree_agb <- ifelse(lgtrees$lifeform == "tree" & lgtrees$genus == "Dipterocarpus", dipt_bas_model_3,

                    ifelse(lgtrees$lifeform == "tree" & lgtrees$genus == "Hopea", hope_bas_model_3,

                    ifelse(lgtrees$lifeform == "tree" & lgtrees$genus == "Shorea", shor_bas_model_3,

                    ifelse(lgtrees$lifeform == "tree" & lgtrees$genus == "Palaquium", pala_bas_model_3,

                    ifelse(lgtrees$lifeform == "tree" & lgtrees$species_group == "Dipterocarp", comm_bas_model_3,

                    ifelse(lgtrees$lifeform == "tree" & lgtrees$species_group == "Other commercial", comm_bas_model_3,

                    ifelse(lgtrees$lifeform == "tree" & lgtrees$species_group == "Fruit tree", mixe_bas_model_3,

                    ifelse(lgtrees$lifeform == "tree" & lgtrees$species_group == "Pioneer", mixe_bas_model_3,

                    ifelse(lgtrees$lifeform == "tree" & lgtrees$species_group == "Other", mixe_bas_model_3, NA)))))))))


#applying models for estimating liana biomass

lgtrees$liana_agb <- ifelse(lgtrees$lifeform=="liana", liana_model, NA)


#applying models for estimating dead wood biomass

lgtrees$dead_agb <- ifelse(!is.na(lgtrees$lifeform=="dead" & lgtrees$snag_height>0), lgtrees$dead_wood_mass, lgtrees$dead_wood_mass_est)
```

```
lgtrees$dead_agb <- lgtrees$dead_agb / 1000 #convert dead AGB to g from kg


#calculating total AGB

lgtrees$tot_large_biomass <- rowSums(lgtrees[, c("liana_agb", "tree_agb", "
dead_agb")], na.rm = TRUE)


#carbon content

CC=0.47


#constant for carbon per hectare

constant_CC = (10000 / (60 * 60)) * CC


#aggregate plot data

plot_agb = aggregate(list(lgtrees[c(31:34)]), by=list(lgtrees$plot), sum, n
a.rm=TRUE)

colnames(plot_agb)[1] <- "Plot"


#calculate plot C

plot_C = plot_agb[, c(2:5)] / 1000 * constant_CC #converts kg AGB to MgC pe
r hectare

plotsum_large = cbind(plot_agb[1], plot_C)


#write CSV

write.csv(plotsum_large,"C:/plotsum_large.csv")
```
